# Draft Genome Sequences of Seven Cronobacter sakazakii Strains Carrying the *mcr* 9.1 Gene Isolated in Chile

**DOI:** 10.1128/MRA.00506-21

**Published:** 2021-07-15

**Authors:** Julio Parra-Flores, Ondrej Holý, Francisca Riffo, Sarah Lepuschitz, Werner Ruppitsch, Stephen Forsythe

**Affiliations:** a Department of Nutrition and Public Health, Universidad del Bío-Bío, Chillán, Chile; b Department of Public Health, Palacký University Olomouc, Olomouc, Czech Republic; c Ñuble Health Service, Ministry of Health, Chillán, Chile; d Austrian Agency for Health and Food Safety, Institute for Medical Microbiology and Hygiene, Vienna, Austria; e foodmicrobe.com, Adams Hill, Keyworth, Nottinghamshire, United Kingdom; University of Maryland School of Medicine

## Abstract

Cronobacter sakazakii is a pathogen that causes severe diseases such as meningitis and necrotizing enterocolitis in infants under 12 months, associated with the consumption of contaminated rehydrated powdered infant formula (PIF). We present seven C. sakazakii genome sequences isolated from PIF and dairy products in Chile in 2017.

## ANNOUNCEMENT

*Cronobacter* is a genus of opportunistic pathogens associated with a number of severe diseases such as meningitis, septicemia, and necrotizing enterocolitis in infants (1). The *Cronobacter* genus includes seven species, of which Cronobacter sakazakii is the one most often associated with disease cases (2, 3). The disease is epidemiologically linked to the consumption of rehydrated powdered infant formula (PIF) that has been contaminated during manufacture or by the reconstitution water (4, 5). In 2017, the Chilean Ministry of Health issued a national and international food alert due to the presence of C. sakazakii in PIF (6).

The C. sakazakii strains were isolated from PIF and dairy products after preenrichment in buffered peptone water (BPW), followed by culturing in *Enterobacteriaceae* enrichment broth (BD Difco, Sparks, MD, USA), isolation on Brilliance chromogenic agar CM 1035 (Oxoid Thermo Fisher, UK), and purification on Trypticase soy agar (BD Difco). The strains were identified using matrix-assisted laser desorption ionization–time of flight (MALDI-TOF) mass spectrometry (Bruker, Billerica, MA, USA). High-quality genomic DNA was obtained from an overnight culture at 37°C on Trypticase soy agar using the MagAttract high-molecular-weight (HMW) DNA kit (Qiagen, Hilden, Germany). Quantification of the input DNA was performed using a Qubit 2.0 fluorometer (Thermo Fisher Scientific, Waltham, MA, USA) and the double-stranded DNA (dsDNA) broad-range (BR) assay kit (Thermo Fisher Scientific). Sequence libraries were generated using the Nextera XT kit (Illumina, Inc., San Diego, CA, USA) and sequenced in paired-end (2 × 300-bp) format on a MiSeq instrument (Illumina, Inc.) as previously described (7). Default parameters were used for all software unless otherwise specified. The raw reads were quality controlled using FastQC v0.11.9. Trimmomatic v0.36 (8) was used to remove the adapter sequences and to trim the last 10 bp of each sequence and sequences with quality scores of <20. The reads were assembled using SPAdes v3.11.1 (9). The contigs were filtered for a minimum coverage of 5× and a minimum length of 200 bp using SeqSphere+ v6.0.0 software (Ridom GmbH, Würzburg, Germany). The whole-genome shotgun (WGS) sequencing of seven C. sakazakii isolates generated 258,038 to 1,347,552 reads, with a coverage ranging from 14× to 85×. The NCBI Prokaryotic Genome Automatic Annotation Pipeline v5.2 (10) identified 4,214 to 4,634 genes, 4,010 to 4,417 coding sequences, 86 to 104 pseudogenes, 101 to 126 RNA genes, and 1 to 3 CRISPR arrays (Table 1). Species identification was performed with a BLAST search against the NCBI 16S rRNA database (10), Mash v1.0 distance analysis (11), and ribosomal multilocus sequence typing (rMLST) (12). The species were characterized using multilocus sequence typing (MLST) (13) extracted from the PubMLST *Cronobacter* database (https://pubmlst.org/organisms/cronobacter-spp) and core genome MLST (cgMLST) using SeqSphere+ v6.0. with default settings as previously described (7, 14). All isolates had >97% good cgMLST targets, sequence type 1 (ST1) and ST83, and clonal complex 1 (CC1) and CC83. Analysis of the genomes using AMRFinderPlus v3.10.5 (15), PlasmidFinder v2.1 (16), and CRISPRCasFinder v2.0.3 (17) revealed that all the isolates carried the intrinsic resistance genes *mcr 9*.1 and *bla*_CSA_ and the plasmids Col440I, Col(pHHAD28), and IncFII(pECLA). All the C. sakazakii strains showed CRISPR arrays, and three strains had both I-E and I-F type arrays.

**TABLE 1:** Genomic information and GenBank accession numbers of seven Cronobacter sakazakii isolates obtained from powdered infant formula and powdered milk

Strain	No. of reads	Genome size (kb)	GC content (%)	Avg coverage (×)	No. of contigs	*N*_50_ (bp)	No. of genes	No. of CDS^*a*^	No. of RNA genes	No. of CRISPR arrays	ST^*b*^/CC^*c*^	Source^*d*^	GenBank accession no.	SRA accession no.
CH42	995,516	4,758	56.59	53	129	263,218	4,601	4,381	95	3	1/1	PIF	JAGYXM000000000	SRR14583050
CH43	1,016,998	4,757	56.61	58	112	263,224	4,583	4,373	92	3	1/1	PIF	JAGYXL000000000	SRR14583049
CH44	1,317,030	4,759	56.60	73	120	309,596	4,587	4,375	89	3	1/1	PIF	JAGYXK000000000	SRR14583048
CH45	1,119,674	4,757	56.60	65	114	322,002	4,598	4,382	90	3	1/1	PIF	JAGYXJ000000000	SRR14583047
CH50	258,038	4,348	57.04	14	252	35,014	4,215	4,010	104	1	83/83	PM	JAGYXI000000000	SRR14583046
CH65	770,710	4,777	56.60	43	174	232,879	4,634	4,417	96	3	1/1	DP	JAGYXH000000000	SRR14583045
CH84	1,347,552	4,761	56.58	85	120	232,569	4,597	4,389	86	3	1/1	PIF	JAGYXG000000000	SRR14583044

a CDS, coding sequences.

b The sequence type (ST) was determined by uploading the genome assemblies to https://pubmlst.org/cronobacter.

c CC, clonal complex.

d PIF, powdered infant formula; PM, powdered milk; DP, dairy product.

### Data availability.

This whole-genome shotgun project has been deposited in DDBJ/ENA/GenBank under the BioProject accession no. PRJNA727616 with the accession no. JAGYXG000000000 to JAGYXM000000000. The version described in this paper is JAGYXG010000000 to JAGYXM010000000. The raw sequence reads have been deposited in the Sequence Read Archive (SRA) under accession no. SRR14583044 to SRR14583050.
